# Proceedings of the Second District Dental Society, N. Y.

**Published:** 1871-02

**Authors:** 


### PROCEEDINGS OF THE SECOND DISTRCT DENTAL SOCIETY, N. Y.

Report of discussions in the Second District Society, held at the office of Dr. Ambler, Brooklyn, January 10th, 1871. Thirty-one present. The meeting was one of the most spirited that has been convened. An afternoon and evening session was held. At six P. M. the Society sat down to a most sumptuous supper, provided by Dr. R. T. Ambler, which was fully applied and appreciated.

The subject for discussion was: "The Method and Propriety of Extirpating Pulps."

Dr. Marvin was the essayist. His paper reviewed the subject strictly from a conservative standpoint.

Dr. Elliott says he does not feel warranted in trying to save pulps that had been inflamed. Thinks if they are once inflamed, they are reduced in their recuperative power. Caps pulps from the ages of fifteen to thirty-five. Prefer Patients of nervous sanguine temperaments. For the last eight months has kept a record. Has had uniform success. To prepare pulps for extirpation, devitalizes with arsenic and creosote. Is not afraid to leave it in the tooth indefinitely. Thinks one application will not necessarily destroy the whole pulp. Acts on the vital principle. Don't know just what that is. Does not think it acts on the circulation. Believes the pulp can be restored to its normal condition of health if partially destroyed. Admires those dentists who endeavor *intelligently* to save inflamed pulps.

Dr. Hill: Believes some pulps can be capped and saved. Would not attempt to save one less than thirty years old. Does not believe a pulp can be restored after an application of arsenic. Can not find anything laid down in books to prove the fact, and has consulted physicians upon this point, without being able to elicit any proof of its being done.

Dr. A. H. Brockway says: Nerve tissue can be reproduced. Referred to cases where teeth have been removed from their sockets, replaced, and assuming their former functions of use and comfort. Does not use *morphia* with arsenic and creosote for devitalizing pulps. Covers the application with cotton and sandarach varnish, to protect the tissues from the arsenic.

Dr. Marvin: Would add wax for a covering, giving greater security from the danger to which the process as well as the tissues are exposed.

Dr. Elmendorf: To make greater security with the stoppings named, he applies the rubber dam, to facilitate the operation.

Dr. A H. Brockway asked if any one present saved all pulps alive? Said Dr. Atkinson *thinks* he does.

Dr. Mills says he *tries* to save *all*. Has not capped a large number. Knows of three that have died. One, a young subject of a low-toned organization. Was inflamed. Was a six-year molar. Pus discharged from it. Treated it with aconite and creosote, alternating according to symptoms. Considered it restored to its normal condition, but it *expired*. Recognizes the fact that some cases are more favorable than others. Does not think because *he* failed, that an *intelligence of a higher order* would necessarily fail. Does not apply *death* to a pulp, knowingly, any more. "Does not give *life*, does not feel that he has any right to take it."

Dr. Marvin saturates bibulous paper with creosote and packs it gently over the exposed pulp. Then applies oxychloride of zinc. Thinks the paper prevents the excruciating effects of the chloride of zinc. Thinks some pulps do live after being capped. He wants to think the profession is making some advance.

Dr. Elliott looks upon the creosote as the agent of success, not the oxy-chloride of zinc cap. Saturates freely with creosote. He adds, that he believes arsenic destroys cell by cell, molecule by molecule; therefore its action could be arrested.

Dr. Marvin believes if this action of arsenic is by the continuity of molecules, its action would continue in its power to destroy. Leaves the arsenic in, twenty-four hours; if not sufficiently devitalized, renews the application until he is able to remove the whole pulp. He added, that he had treated a few pulps by capping with oxy-chloride, had no reports to make. Was not clear in his own mind, and did not know that any one was.

Dr. Hurd said that the trying to save exposed pulps alive by capping, was no new thing. He had gone over the field of experiments in this line, and had decided to destroy all pulps exposed, by the application of arsenic and creosote. Thought the nerve extirpated alive was the safest way, but it was accompanied with so much pain, patients would not submit to it. Lets the arsenic remain eight days. If it remains longer will cause periostitis. Says if the pulp is removed after exposure it is a safe operation; to cap is not. Says oxy-chloride is a cruel application, and *will* destroy the pulp. It may be quiet for a time, but a *volcano* is there, and one day it will show its power, and bring sorrow to those who have capped the pulps. Says a great number of those that have been capped and heralded as successes, have been extracted, because of the mischief they have done. Says the albumenizing effect is only a theory, and is not proved in practice. May save one pulp out of ten; but the nine will scare us out of the practice if we see them again. But says the troublesome ones do not return, they go to another dentist, and complain of the practice. He extirpates and is *always safe.*

Dr. Mills said if it is proved that *one* could be saved, it was evidence to his mind, that *many more* can be, based upon the *same principles* on which the one was saved. And believed it to be our *duty* to seek the *light*, and walk in it *willingly* and *faithfully.*

